# Retroperitoneal pheochromocytoma: Unsual presentation and atypical location

**DOI:** 10.1016/j.ijscr.2021.106248

**Published:** 2021-07-27

**Authors:** Carlos Eduardo Rey Chaves, Daniela Ayala, Gabriel García, Danny Conde Monroy, Juan Carlos Sabogal Olarte

**Affiliations:** a Hepatobiliary and Pancreatic Surgery, Hospital Universitario Mayor Méderi, Colombia; b Universidad el Rosario, Colombia; c Hepatobiliary and Pancreatic Surgery, HPB Surgery Department, Hospital Universitario Mayor Méderi, Colombia

**Keywords:** Pheochromocytoma, Surgery, Chromogranin A, Hypertension

## Abstract

**Introduction and importance:**

Pheochromocytomas are rare tumors (0.1–2% of incidence), arising from the chromaffin cells in the sympathoadrenal system. Approximately 85% of the times are localized in the adrenal medulla; therefore, could be placed extra adrenal in 15% of the population. 10–30% of the cases could be asymptomatic. Classic symptoms vary from palpitations, tachycardia, hypertension.

**Case presentation:**

Case report of a 37-year-old female patient presented with diffuse abdominal pain, with any associated symptoms. Contrast computed tomography was performed; a retroperitoneal mass was found, contacting the third portion of the duodenum. Intraoperative hypertensive crisis was documented with the manipulation of the mass. Octreotide infusion was administered with the normalization of the clinical condition. Patients do not present any postoperative morbidity after 90 days. Pathology reports chromaffin cells concluding pheochromocytoma.

**Discussion:**

Pheochromocytomas are rare tumors with an annual incidence between 3 and 8 cases per million population per year in some series of cases. In general terms prevalence rounds 0.1–0.6% of patients with hypertension. Surgical management is the definitive treatment for pheochromocytoma benign or malign. Morbidity described in literature reaches 40% with 20% of mortality in some series of cases. In our patient we do not present postoperative complications.

**Conclusion:**

Intraoperative hypertension is a clinical and surgical challenge, not only for the surgeon, also anesthesiology. Pheochromocytoma it's a complex entity and could be silent in until 30% of the cases, should be suspected in all neuroendocrine retroperitoneal tumors. Multidisciplinary approach with anesthesia, endocrinology and surgery department is mandatory to have good postoperative outcomes.

## Highlights

• Retroperitoneal location of pheochromocytoma it's uncommon, in literature, only 4 cases are reported to the date.
• Silent neuroendocrine tumors could represent an intraoperative challenge developing hypertensive crisis.
• Multidisciplinary management of neuroendocrine tumors impact in postoperative outcomes

## 1 Introduction

Pheochromocytoma (PHEO) is a rare tumor originated from the neural crest and located in the adrenal medulla chromaffin cells in the 80% of the cases, 20% are located outside the adrenal medulla in other organs/locations with neural crest cells [1]. This origin of the tumors is related with the production and secretion of catecholamines and represents between 15 and 20% of all the catecholamine productive neoplasms [2]. In patients with hypertension, the prevalence of PHEO is around 0.1–0.6%, with no difference of presentation between sex, and are most frequent in the fourth - fifth decade of life [1]. The majority of the cases are symptomatic (70% approximately) [3] but a good proportion of the patients could present a silent entity [3].

.Clinical features vary from hypertension (49%), headaches (51.9%), diaphoresis (48.8%), flushing (35%) and abdominal pain in a less proportion with 19.5% [1] in some patients could appear classic triad of headaches, palpitations, and sweating; however, in the literature are described a proportion of 25% of PHEO were never diagnosed during life [4]. This varied clinical presentation is the reason that PHEO is called “Great mimic “, and could delay the diagnosis, and the management [5].

.Pheochromocytoma it's a rare entity as we noted, the localization of the tumor is also an important fact. Literature reports a low number of cases of extra-adrenal PHEO, and in most of the cases are located below the diaphragm in the organ of Zuckerkandl [6]. Retroperitoneal presentations of these tumors are very rare, in the literature only 4 tumors are reported in the international literature [7], [8], [9], [10], [11], [12].

Surgical removal of PHEO is the gold standard management for fit patients, either adrenal or extra-adrenal localization [13]. Surgical procedures could be associated with paroxysmal events of hypertension in patients with this comorbidity even if they have any pre-medication in 25% of the cases [13]. Intraoperative unexpected hypertension may lead to catastrophic events with multiorgan impact in patients [14], in this case explained due to the liberation of catecholamines, and represent not only a surgical, also an anesthesia challenge [15], [16]. Interestingly the clinical presentation in the surgical procedure in the 30% of the cases its related with the manipulation of the mass, or the ligation of tumor vasculature [16], [17], [18]. .

## 2 Clinical case

### 2.1 Diagnosis

A female 37-year-old patient, with clinical history of papillary thyroid cancer (Follicular and onchocitic variation) entered the emergency room with 2 days of abdominal pain at mesogastrium, associated with diarrhea. No history of hypertension or tachycardia, also patient don't have history of drug use.

Physical exam with abdominal tenderness in mesogastrium and left iliac fossa irradiated to the back, with no peritoneal irritation. Arterial pressure 125/70 mmHg, heart rate 79 bpm. Normal blood count, CRP (C-Reactive-Protein) - 75 mg/L -; liver enzymes with alkaline phosphatase value of 150 UI/L; pregnancy test negative.

**Image 1:**
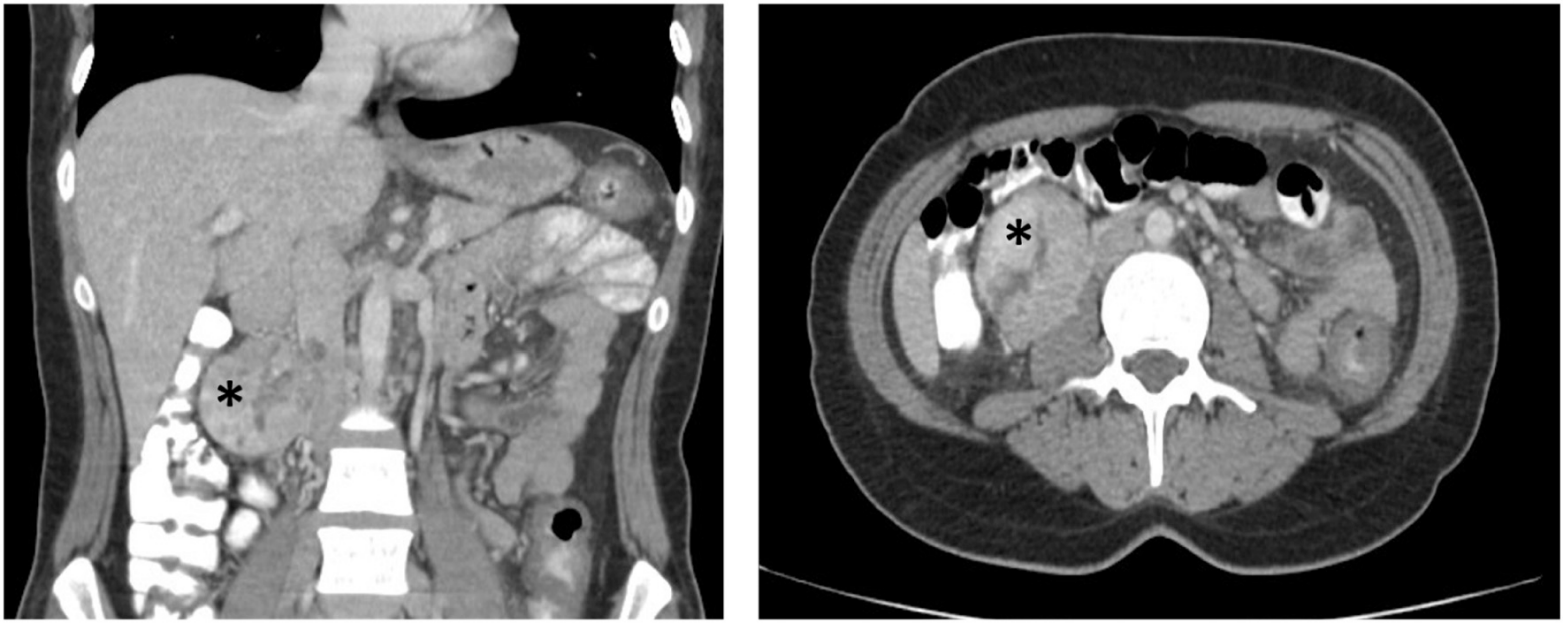
A. Coronal view in abdomen CT (*tumoral mass)/B. Sagital view in abdomen CT (*tumoral mass).

**Image 2:**
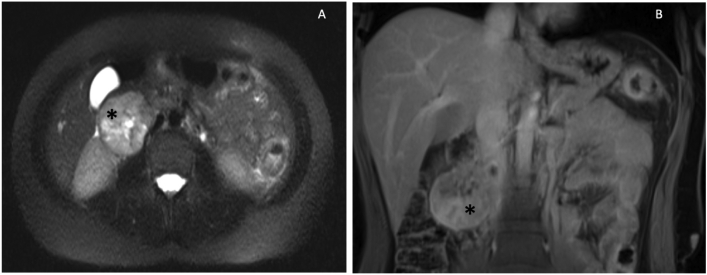
A. Sagital view in MRI (*tumoral mass). B. Coronal view in MRI (*tumoral mass).

We perform an initial abdominal computed tomography (CT), with a 59 × 47 × 58 CMS mass adjacent to the second duodenal portion with calcifications (Image 1). Abdominal magnetic resonance (MRI) was also performed; MRI showed a solid mass with necrotic-cystic central component; suspected differential diagnosis comprises a neuroendocrine tumor of the head of pancreas, duodenal gastrointestinal stromal tumor (GIST), or ganglionar conglomerate (Image 2). Taking in count the localization near to biliary tract and duodenum, hepato-pancreato-billiary surgeon was requested in order to continue the management of the patient.

Esophagogastroduodenoscopy was performed to evaluate duodenal lumen, with any findings.

With a clinical suspected neuroendocrine tumor, biochemical markers such as chromogranin A and 5-Hydroxyindoleacetic acid were requested. Chromogranin A was positive (275 ng/dL), and 5HAA was negative in 24 h urine recollection (2.7 mg/24 h in a 3180 volume).

Anesthesia, and endocrine services were requested for preoperative assessment, no history of high values of arterial pressure was found, no paroxysmal tachycardia was found after 24 h of vigilance. Initially, metanephrine production was evaluated with negative results. (Normetanephrines 0.78; and free metanephrine <0.20). To confirm our results, we search for any confounder factor that could interfere with the analysis, such as beta-blockers medication, plasmatic concentrations of metanephrines was performed, as urine evaluation, was negative. With these findings the patient was considered a surgical candidate to resection, patient was notified, and accepts the medical decision.

### 2.2 Intraoperative findings

**Image 3:**
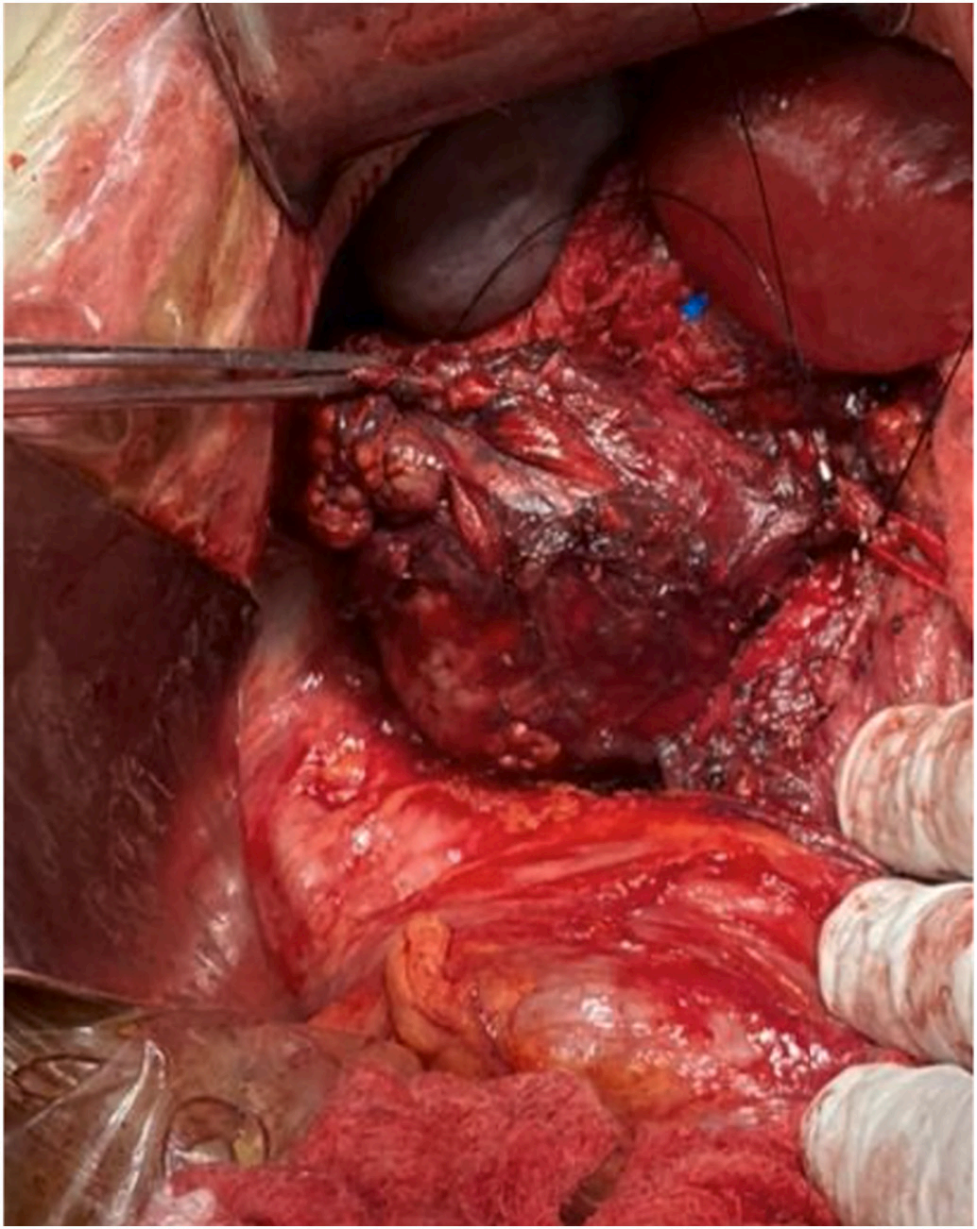
Tumor exposition and vascular ligation.

We identify an important mass in contact with the second duodenal portion. Performing a Kocher maneuver, we start tumoral dissection, finding right adrenal compromise (Image 3). During the manipulation of the mass, patients present a hypertensive crisis with Arterial pressure (AP) 189/94 with Mean arterial pressure (MAP) 125 mmHg. To perform and adequate control of arterial pressure, anesthesiologist start and initial dose of diuretics (Furosemide) with any change in the PA, for that reason, beta-blockers was needed, after two bolus administration of Labetalol, arterial pressure was normalized.

Identifying the renal hilum, we carefully dissect the tumor, and perform ligation of the vasculature.

After the removal of the mass, patients present a hemodynamic compromise due to hypotension, with PAM 40 mmHg, and anesthesiologists need to start vasopressors to control arterial pressure.

Surgical time was 95 min, with 100 mL of bleeding. Mean value of arterial pressure during the surgery was 180/90 mmHg, with a range between 50/30 mmHg and 189/94 mmHg.

### 2.3 Postoperative care

In the immediate postoperative time, the patient needed a critical care unit due to the requirement of hemodynamic support with vasopressors (Noradrenaline). After 24 h patients do not tolerate suspension of the support, for that reason, the endocrinology department considers that could be explained by the neuroendocrine production (taking in count the positive value of Chromogranin A) and starts octreotide infusion for 24 h. (0.1 mg + 450 cm^3^ of saline solution: Initial bolus of 100 cm^3^ and continue 50 cm^3^/h until blend finish).

After the infusion of somatostatin agonist, patients tolerate the suspension of vasopressors obtaining mean value of PA of 60 mmHg, total ICU stay was 2 days. In-hospital total stay was 5 days.

No complications were observed after 90 postoperative days.

### 2.4 Pathology and histochemical markers reports

Tumor with solid nests of polygonal cells, separated by fibro-capillary septa, positive for chromogranin, sinaptofisin, CD 56, S100 in sustentacular cells. Ki-67 index 2–3%, these being compatible with the pathological anatomy of pheochromocytoma.

Taking in count the association with the previous surgical intervention for a papillary thyroid tumor, we suspect a possible genetic alteration, for that reason genomic analysis was performed; principal mutations such as *EGLN1*, *FH*, *K1F1B*, *MEN1*, *NF1*, *RET*, *SDHC*, *SDHD*, *and TMEM127*, was negative; any genetic mutations was detected.

## 3 Discussion

Pheochromocytomas are rare tumors with an annual incidence between 3 and 8 cases per million population per year in some series of cases [19]. In general terms prevalence rounds 0.1–0.6% of patients with hypertension [20]. Besides clinical presentation with hypertension, tachycardia, or flushing, approximately 0.1–0.5% of the cases are misdiagnosed and recognized in autopsy; and in almost 5% of the cases are discovered in an incidental way in abdominal imaging studies such as MRI, or CT scan because in almost 30% of the cases, patients remains clinically silent during the life [20], [21], [22]. In 70–80% of the cases diagnosis is made during the fourth–fifth decades. PHEO, it's responsible for the 0.1–0.9% of the cases of hypertension [18].

Clinical aspects of pheochromocytoma are variable because signs and symptoms are secondary to the endocrine actions of adrenal products such as epinephrine, norepinephrine and dopamine. It's known as “great mimic” [20]. In recent literature the most relevant symptoms are described as hypertension (80.7%), headache (60.4%), palpitations (59.3%); diaphoresis (50.1%) however other clinical presentations are described, as nausea, vomiting, heat intolerance, blurred vision, anxiety, and abdominal pain [18], [21]. Literature reports nearly 30% of the patient's diagnosis is made as an incidental finding, with minimal clinical signs, and any biochemical findings [18].

The diagnosis it's a summary of clinical presentation and suspected neuroendocrine tumor, biochemical tests, and radiology findings [20]. Classical biochemical tests are the measurement of urinary and plasma catecholamines and free metanephrines [20]. Also, as a neuroendocrine tumor, the production of chromogranin A is present, for that reason elevation of this marker is related to neuroendocrine mass because this enzyme comprises 40% of the production of the chromaffin cells. Abnormal values of chromogranin A are related with the presence of neuroendocrine tumors with high sensitivity and specificity [23].

Abdominal CT scan is the preferred initial radiology diagnostic approach to these tumors with almost a 85% of sensitivity, could make the diagnosis with arterial and venous phases, and localizing the tumor with precision [24], [25]. Also, MRI scans have a high sensitivity with 95%, and is recommended with CT in the preoperative staging [25]. CT and MRI also define with precision the adequate localization of the mass [25]; in most of the cases the “typical” location is adrenal, the extra-adrenal was documented occasionally [25], [26], [27], [28], [29]. In the literature, retroperitoneal location of these tumors it's not common, of 11 cases reported extra-adrenal, just 4 are in this localization [7], [8], [9]; one important fact is that in all of the cases these tumors appears in young patients (less than 40 years old), with no sex preference [6].

When diagnosis is made, the objective of the treatment it's to control the catecholamines production, frequently with B-agonist, avoiding cardiovascular clinical representation (Hypertension, tachycardia) [26]. In our case, the clinical, and surgical challenge is represented with the silent presentation, and intraoperative crisis of hypertension that impacts the hemodynamic state of the patient with requirement of vasopressors. As the diagnosis was not established the patient resisted many of the medical therapies, as reported in the literature [26]. In our case, the fast assessment of the mass, and vasculature ligation prevents persistent hypertension.

Surgical management is the definitive treatment for pheochromocytoma benign or malign. Morbidity described in literature reaches 40% with 20% of mortality in some series of cases. In our patient we do not present postoperative complications [27]. In the literature, are described important principles to the tumoral resection of these lesions. The most important points in the surgical resection, are the minimal tumor manipulation (avoiding hypertensive crisis) and quick vascular supply of the tumor to restrict the endocrine pass to the plasma and controlling catecholamine symptoms. Open anterior approach is preferred in some cases because of the clear exposure of the tumor and near strictures [27]. In our case, the open approach was preferred because of the contact with the duodenum.

## 4 Conclusion

Pheochromocytoma is a rare and complex entity; both, for diagnosis and management. Atypical locations should not dismiss the diagnosis. Retroperitoneal presentation it's extremely rare with only 4 reports in the literature. Surgical intervention should be performed carefully to avoid intraoperative complications. Silent presentation of these tumors is frequent, reaching almost 30%. Multidisciplinary approach leads positive outcomes.

## Funding

This research did not receive any specific grant from funding agencies in the public, commercial, or not-for-profit sectors.

## Sources of funding

No funding.

## Ethical approval

Following Helsinki declaration, ethical committee approve the production of the paper.

## Consent

Written informed consent was obtained from the patient for publication of this case report and accompanying images. A copy of the written consent is available for review by the Editor-in-Chief of this journal on request.

## Author contribution

Carlos Rey: Manuscript, bibliography research

Juan C. Sabogal: Manuscript edition,

Danny Conde: Manuscript edition

Daniela Ayala: Manuscript, bibliography research

Gabriel Garcia: Manuscript, bibliography research

## Registration of research studies

N/A.

## Guarantor

Carlos Eduardo Rey Chaves.

## Provenance and peer review

Not commissioned, externally peer-reviewed.

## Declaration of competing interest

Authors do not declare any conflicts of interest.

## Figures and Tables

**Image 4:**
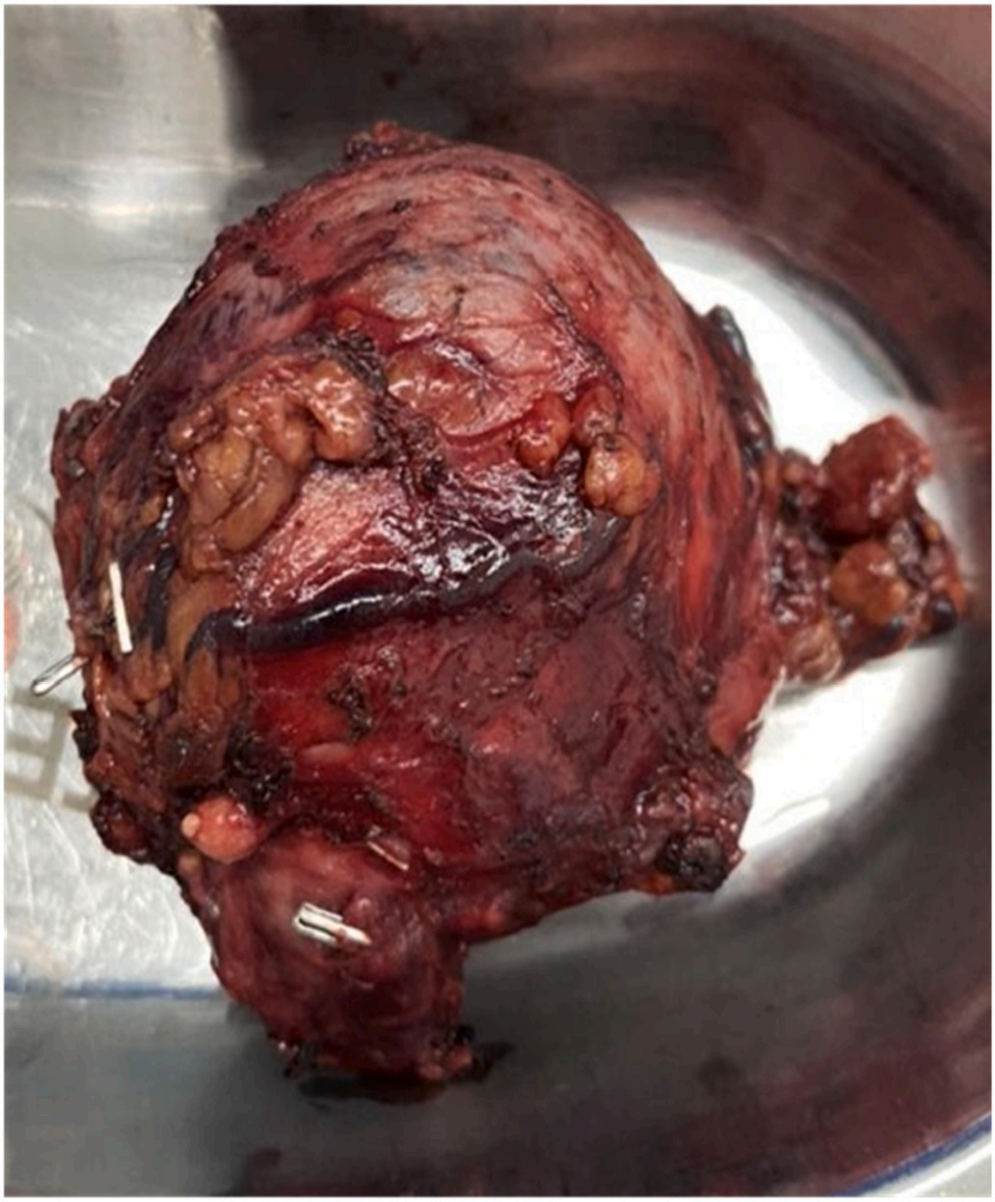
Surgical piece.

## References

[1] Cerqueira A., Seco T., Costa A., Tavares M., Cotter J. (May 5 2020). Pheochromocytoma and paraganglioma: a review of diagnosis, management and treatment of rare causes of hypertension. Cureus.

[2] Falhammar H., Kjellman M., Calissendorff J. (Jan 2018). Initial clinical presentation and spectrum of pheochromocytoma: a study of 94 cases from a single center. Endocr. Connect..

[3] Shen S.J., Cheng H.M., Chiu A.W., Chou C.W., Chen J.Y. (Jan 2005). Perioperative hypertensive crisis in clinically silent pheochromocytomas: report of four cases. Chang Gung Med. J..

[4] Khorram-Manesh A., Ahlman H., Nilsson O., Oden A., Jansson S. (2004). Mortality associated with pheochromocytoma in a large Swedish cohort. Eur. J. Surg. Oncol..

[5] Amar L., Servais A., Gimenez-Roqueplo A.P., Zinzindohoue F., Chatellier G., Plouin P.F. (2005). Year of diagnosis, features at presentation, and risk of recurrence in patients with pheochromocytoma or secreting paraganglioma. J. Clin. Endocrinol. Metab..

[6] Hu J., Wu J., Cai L., Jiang L., Lang Z., Qu G., Liu H., Yao W., Yu G. (2013 Apr). Retroperitoneal composite pheochromocytoma-ganglioneuroma : a case report and review of literature. Diagn. Pathol..

[7] Chen C.H., Boag A.H., Beiko D.T., Siemens D.R., Froese A., Isotalo P.A. (2009). Composite paraganglioma-ganglioneuroma of the urinary bladder: a rare neoplasm causing hemodynamic crisis at tumour resection. Can. Urol. Assoc. J..

[8] Gong J., Wang X., Chen X., Chen N., Huang R., Lu C., Chen D., Zeng H., Zhou Q. (2010). Adrenal and extra-adrenal nonfunctioning composite pheochromocytoma/paraganglioma with immunohistochemical ectopic hormone expression: comparison of two cases. Urol. Int..

[9] Hirasaki S., Kanzaki H., Okuda M., Suzuki S., Fukuhara T., Hanaoka T. (2009). Composite paraganglioma-ganglioneuroma in the retroperitoneum. World J. Surg. Oncol..

[10] Pytel P., Krausz T., Wollmann R., Utset M.F. (2005). Ganglioneuromatous paraganglioma of the cauda equina–a pathological case study. Hum. Pathol..

[11] Tohme C.A., Mattar W.E., Ghorra C.S. (2006). Extra-adrenal composite pheochromocytoma-ganglioneuroma. Saudi Med. J..

[12] Yoshimi N., Tanaka T., Hara A., Bunai Y., Kato K., Mori H. (1992). Extra-adrenal pheochromocytoma-ganglioneuroma. A case report. Pathol. Res. Pract..

[13] Lenders Jacques W.M., Duh Quan-Yang, Eisenhofer Graeme, Gimenez-Roqueplo Anne-Paule, Grebe Stefan K.G., Murad Mohammad Hassan, Naruse Mitsuhide, Pacak Karel, Young William F. (1 June 2014). Pheochromocytoma and paraganglioma: an endocrine society clinical practice guideline. J. Clin. Endocrinol. Metab..

[14] Hariskov S., Schumann R. (Jan 2013). Intraoperative management of patients with incidental catecholamine producing tumors: a literature review and analysis. J. Anaesthesiol. Clin. Pharmacol..

[15] Pacak K. (2007). Approach to the patient. Preoperative management of the pheochromocytoma patient. J. Clin. Endocrinol. Metab..

[16] Kenny L., Rizzo V., Trevis J., Assimakopoulou E., Timon D. (Jul-Sep 2018). The unexpected diagnosis of phaeochromocytoma in the anaesthetic room. Ann. Card. Anaesth..

[17] Hariskov S., Schumann R. (Jan 2013). Intraoperative management of patients with incidental catecholamine producing tumors: a literature review and analysis. J. Anaesthesiol. Clin. Pharmacol..

[18] Farrugia F., Martikos G., Tzanetis P., Charalampopoulos A., Misiakos E., Zavras N., Sotiropoulos D. (2017). Pheochromocytoma, diagnosis and treatment: review of the literature. Endocr. Regul..

[19] Ariton M., Juan C.S., AvRuskin T.W. (2000). Pheochromocytoma: clinical observations from a 546 Brooklyn tertiary hospital. Endocr. Pract..

[20] Sbardella E., Grossman A.B. (2019). Pheochromocytoma: an approach to diagnosis. Best Pract. Res. Clin. Endocrinol. Metab..

[21] Soltani A., Pourian M., Davani B.M. (2016 Mar). Does this patient have pheochromocytoma? A systematic review of clinical signs and symptoms. J. Diabetes Metab. Disord..

[22] Mantero F., Terzolo M., Arnaldi G., Osella G., Masini A.M., Ali A. (2000). A survey on adrenal 548 incidentaloma in Italy. study group on adrenal tumors of the italian society of 549 endocrinology. J. Clin. Endocrinol. Metab..

[23] Mansmann G., Lau J., Balk E., Rothberg M., Miyachi Y., Bornstein S.R. (2004). The clinically 551 inapparent adrenal mass: update in diagnosis and management. Endocr. Rev..

[24] Yang X., Yang Y., Li Z., Cheng C., Yang T., Wang C., Liu L., Liu S. (2015). Diagnostic value of circulating chromogranin a for neuroendocrine tumors: a systematic review and meta-analysis. PLoS One..

[25] Blake M.A., Boland G.W., Bílek R., Vlcek P., Šafarík L., Michalský D., Novák K., Dušková J., Václavíková E., Widimský J., Zelinka T., Humana Press Inc (2009). Adrenal maging. Chromogranin A in the Laboratory Diagnosis of Pheochromocytoma and Paraganglioma. Cancers (Basel).

[26] Bennett B., Johnson D., Panakos A., Rozenberg A. (2017 Nov 6). Unsuspected pheochromocytoma incidentally found on chest CT. Radiol. Case Rep..

[27] Kline J.P. (2010 Aug). Use of clevidipine for intraoperative hypertension caused by an undiagnosed pheochromocytoma: a case report. AANA J..

[28] Hanna N.N., Kenady D.E., Holzheimer R.G., Mannick J.A. (2001). Pheochromocytoma. Surgical Treatment: Evidence-Based and Problem-Oriented.

[29] Khan A.N., Solomon S.S., Childress R.D. (2010). Composite pheochromocytoma-ganglioneuroma: a rare experiment of nature. Endocr. Pract..

